# Apoptosis-Related Gene-Mediated Cell Death Pattern Induces Immunosuppression and Immunotherapy Resistance in Gastric Cancer

**DOI:** 10.3389/fgene.2022.921163

**Published:** 2022-07-05

**Authors:** Xiaolu Yuan, Jun Zhou, Liping Zhou, Zudong Huang, Weiwei Wang, Jiasheng Qiu, Qiangbang Yang, Chaohao Zhang, MingHui Ma

**Affiliations:** ^1^ Department of Pathology, Maoming People’s Hospital, Maoming, China; ^2^ Department of Gastrointestinal Surgery, Maoming People’s Hospital, Maoming, China; ^3^ Department of Endoscopy Center, The No.6 People’s Hospital of Benxi, Liaoning, China

**Keywords:** apoptosis-related genes, tumor microenvironment, gastric cancer, immunotherapy, scoring system

## Abstract

**Background:** Apoptosis is a type of cell death, which can produce abundant mediators to modify the tumor microenvironment. However, relationships between apoptosis, immunosuppression, and immunotherapy resistance of gastric cancer (GC) remain unclear.

**Methods:** Gene expression data and matching clinical information were extracted from TCGA-STAD, GSE84437, GSE34942, GSE15459, GSE57303, ACRG/GSE62254, GSE29272, GSE26253, and IMvigor210 datasets. A consensus clustering analysis based on six apoptosis-related genes (ARGs) was performed to determine the molecular subtypes, and then an apoptosisScore was constructed based on differentially expressed and prognostic genes between molecular subtypes. Estimate R package was utilized to calculate the tumor microenvironment condition. Kaplan-Meier analysis and ROC curves were performed to further confirm the apoptosisScore efficacy.

**Results:** Based on six ARGs, two molecular subgroups with significantly distinct survival and immune cell infiltration were identified. Then, an apoptosisScore was built to quantify the apoptosis index of each GC patient. Next, we investigated the correlations between the clinical characteristics and apoptosisScore using logistic regression. Multivariate Cox analysis shows that low apoptosisScore was an independent predictor of poor overall survival in TCGA and ACRG datasets, and was associated with the higher pathological stage. Meanwhile, low apoptosisScore was associated with higher immune cell, higher ESTIMATEScore, higher immuneScore, higher stromalScore, higher immune checkpoint, and lower tumorpurity, which was consistent with the “immunity tidal model theory”. Importantly, low apoptosisScore was sensitive to immunotherapy. In addition, GSEA indicated that several gene ontology and Kyoto Encyclopedia of Genes and Genomes items associated with apoptosis, several immune-related pathways, and JAK–STAT signal pathway were considerably enriched in the low apoptosisScore phenotype pathway.

**Conclusion:** Our findings propose that low apoptosisScore is a prognostic biomarker, correlated with immune infiltrates, and sensitivity to immunotherapy in GC.

## Introduction

Gastric cancer (GC) is the fifth reason for incidence and fourth in mortality among all cancers (Siegel et al., 2021). Due to delayed diagnosis and less effective treatment in some cases, the overall survival rate is poor (Smyth et al., 2020). PD-1/PD-L1 inhibitors profoundly switched the treatment landscape of GC (Joshi and Badgwell, 2021; Li et al., 2021). The ATTRACTION 2 trial of patients with unresectable advanced or recurrent GC using navuzumab showed a client remission rate of 11.2% and demonstrated extended overall survival (OS) (Satoh et al., 2020). However, while PD-1/PD-L1 inhibitors are a promising treatment for patients with advanced GC, their response rate is limited and the development of new strategies to maximize the efficacy of ICI is necessary (Kono et al., 2020; Kole et al., 2022). Seeking effective biomarkers to screen patients for immunotherapy is essential to optimize the treatment strategy for GC and improve the prognosis of patients.

Apoptosis is an active and procedural death process of cells in the body, which is essential to preserve the homeostasis of the intracellular environment. When cells receive apoptosis signals, initiation caspases are activated through different signal pathways, and effecting Caspases are activated, and then related substrates are degraded, finally leading to cell apoptosis (D'Arcy, 2019). Apoptosis is associated with the occurrence and development of GC (Frejlich et al., 2013). Different drugs have different mechanisms to regulate apoptosis: benzoxanthone compounds regulating the Bcl-2 related protein and the Bcl-2 protein proportion block induced gastric cancer cell apoptosis (Fu et al., 2022), paclitaxel can be through the regulation of p53 gene-mediated apoptosis induced by cell signal transduction pathways (Niapour and Seyedasli, 2022), cisplatin can also through the apoptosis induced by regulating the cell cycle (Guo et al., 2022). Different drugs can copy a cell block in different stages, causing cells to fail to divide properly and die.

Apoptosis also can assist in the regulation of cancer immunity as both the cell itself and environmental factors can affect the apoptosis of GC cells; for example, T cells and NK cells can induce the apoptosis of gastric cancer cells through perforation or Fas pathway (Kume et al., 1999). At the same time, gastric cancer cells can also evade immune monitoring through a variety of mechanisms, resulting in reduced immune quantity, weakened ability of DC cells to present tumor antigen, and inhibited activation of initial T cells (Li et al., 2019). The apoptosis rate of T lymphocytes in GC patients based on Fas/Fasl pathway was significantly increased, suggesting that immune escape in GC cells was related to the up-regulation of Fas ligand expression and t-lymphocyte apoptosis. T cells, NK cells, and DC cells all express Fas receptor after activation, so it is assumed that GC cells may induce apoptosis of the above immune cells through Fas/Fasl pathway by up-regulating Fas ligand (Katoh et al., 2000).

**FIGURE 1:**
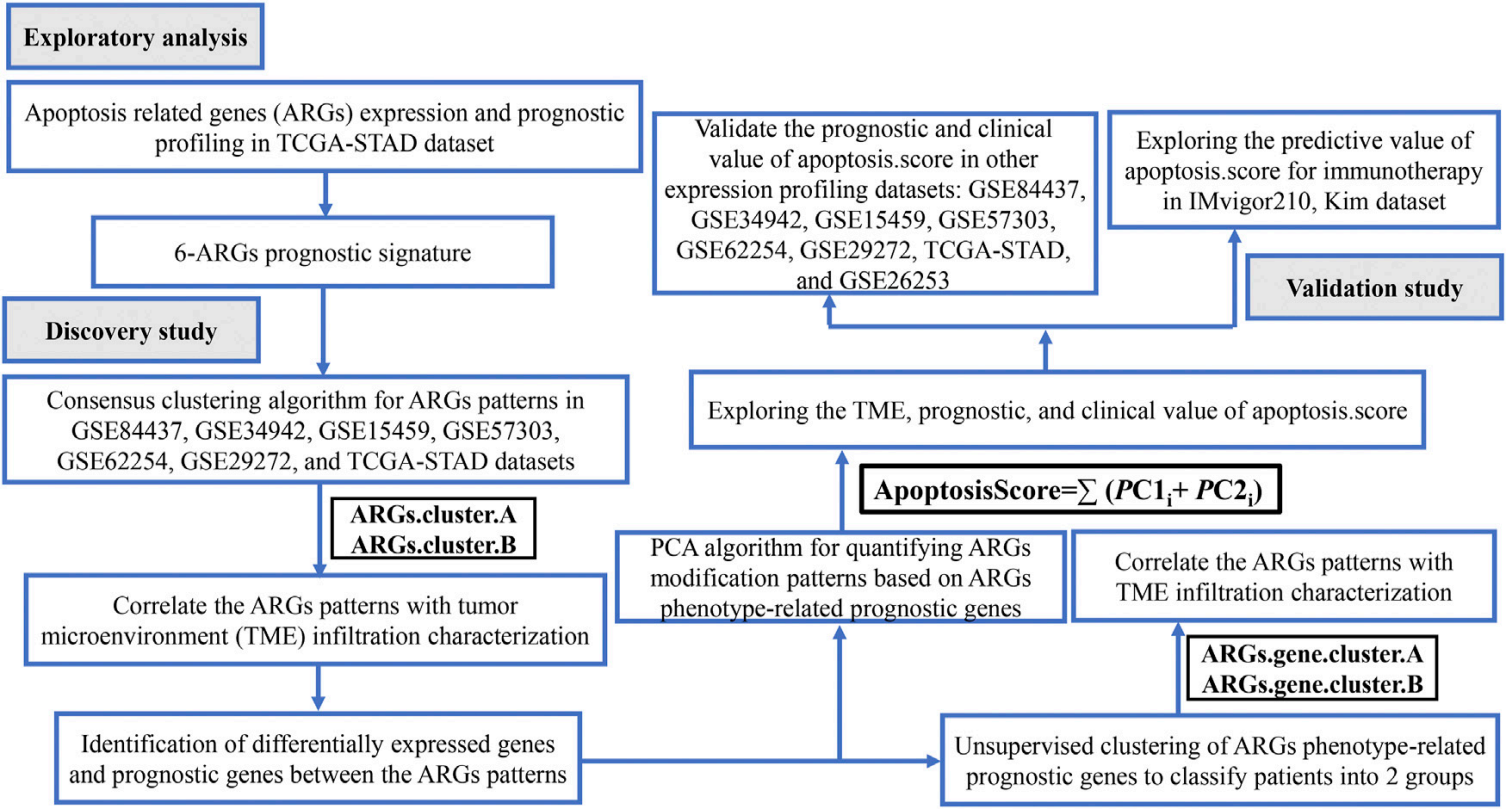
Flow chart of the study.

Workflow Figure 1 illustrates the workflow of our analysis. We first present a comprehensive analysis of the apoptosis-related gene (ARG) expression and prognostic profiling in TCGA-STAD dataset, and found that six differentially expressed and prognostic ARGs were identified. Further, GC samples were classified as two molecular subtypes based on the six ARGs and further validated by KM plotter survival plot. To further elucidate the potential functions of distinct molecular subtypes, we identified 3,083 differentially expressed genes (DEGs) and prognostic genes between the two molecular subtypes and performed functional enrichment analysis on these related genes. We also investigated the correlations between the immune cell infiltration and the two molecular subtypes. Next, GC samples were classified as two molecular subtypes based on the 3,083 genes and further validated by the KM plotter survival plot. Then, an apoptosisScore was constructed based on 3,083 genes. We also examined the correlations between the clinical characteristics and immune cell infiltration and high and low apoptosisScore groups. Finally, we evaluated the accuracy of this novel apoptosisScore and prognostic differences between high- and low apoptosisScore GC patients and analyzed the sensitivity of high- and low-apoptosisScore groups of GC patients to immunotherapy. This novel apoptosisScore model not only accurately predicts the prognosis of GC patients but also offers new insights into the heterogeneity of immunotherapy in GC patients.

## Materials and Methods

### Data Source and Preprocessing

Eight independent GC cohorts were studied in the present study. For the TCGA dataset, mRNA expression files were acquired from the Genomic Data Commons Data Portal (https://portal.gdc.cancer.gov/), and corresponding clinicopathologic data were gained from the cbioportal website (https://www.cbioportal.org/). After removing incomplete data from the survival analysis, data of 350 GC patients complying with the requirements were obtained. Data for the GSE84437 (*N* = 431) (Yoon et al., 2020), GSE34942 (*N* = 56) (Chia et al., 2015), GSE15459 (*N* = 191) (Ooi et al., 2009), GSE57303 (*N* = 70) (Qian et al., 2014), ACRG/GSE62254 (*N* = 300) (Cristescu et al., 2015), GSE29272 (*N* = 126) (Wang et al., 2013), and GSE26253 (*N* = 432) (Lee et al., 2014) were obtained from Gene expression omnibus (GEO) genomics data repository (https://www.ncbi.nlm.nih.gov/geo/). IMvigor210 CoreBiologies data was downloaded using the R package provided by the following website (https://www.nature.com/articles/nature25501) (Mariathasan et al., 2018). The detail was shown in Table 1. R (version 4.0.5) was used to conduct dataset processing and further analysis with R Bioconductor packages.

**TABLE 1:** Basic information of series used in this study.

Series Accession Numbers	Platform Used	No. of Input Patients	Region	Survivval outcome	Data Usage	PMID
GSE84437 (GPL6947)	Illumina HumanHT-12 V3.0 expression beadchip	431	Korea	OS	Training; Internal validation	32,293,340
GSE34942 (GPL570)	Affymetrix Human Genome U133 Plus 2.0 Array	56	Singapore	OS	Training; Internal validation	25,053,715
GSE15459 (GPL570)	Affymetrix Human Genome U133 Plus 2.0 Array	191	Singapore	OS	Training; Internal validation	19,798,449
GSE57303 (GPL570)	Affymetrix Human Genome U133 Plus 2.0 Array	70	China	OS	Training; Internal validation	24,935,174
ACRG/GSE62254 (GPL570)	Affymetrix Human Genome U133 Plus 2.0 Array	300	Asia	OS	Training; Internal validation	25,894,828
GSE29272 (GPL96)	Affymetrix Human Genome U133A Array	126	United States	OS	Training; Internal validation	23,717,493
GSE26253 (GPL8432)	Illumina HumanRef-8 WG-DASL v3.0	432	Korea	RFS	External validation	24,598,828
TCGA-STAD	Illumina RNAseq	350	NA	OS	Training; Internal validation	
IMvigor210	Illumina RNAseq	348	NA	OS	External validation	29,443,960
Kim cohort	Illumina RNAseq	45	Korean	NA	External validation	30,013,197

TCGA: the cancer genome atlas; STAD: stomach adenocarcinoma; ACRG: asian cancer research group; OS: overall survival; RFS: relapse free survival.

### Expression and Prognostic of Apoptosis-Related Genes

By reviewing the previous literature, we identified 92 ARGs (Supplementary Table S1). The expression landscape of ARGs was generated with “limma” and “reshape2” package (|log FC|≥ 1.0 and adj. *p* < 0.05) (Ritchie et al., 2015). The hazard ratio and 95% confidence intervals were calculated by the Cox proportional hazard regression model (Fisher and Lin, 1999).

### Consensus Cluster Analysis

We performed an unsupervised cluster analysis of OS samples by ConsensusClusterPlus R package to identify different subtypes. In this process, the number of clusters was set between 2 and 10, and then the samples were classified using consistent clustering (Wilkerson and Hayes, 2010). After screening for the most optimal subtype classification, OS patients were divided into different subtypes and survival analysis was performed on the clustered samples by the Survival R software package, using log-rank test statistics and plotting Kaplan-Meier curves to analyze the survival differences between subtypes. Subsequently, differential genes of different subtypes were screened by limma R package. Differential genes met adj. *p* < 0.05 and |log2FoldChange|>1.

### Construction of apoptosisScore

Next, we used the principal component analysis (PCA) method to quantify apoptosis-related subtypes of individual patients (David and Jacobs, 2014). An apoptosisScore for each patient was calculated according to the following formula:

$$\mathrm{ApoptosisScore}=\text{Σ}\left(PC1_{i}+PC2_{i}\right),$$

where *i* is the TPM value of each screened gene.

### ESTIMATE Algorithm

The ESTIMATE algorithm, which assessed stromal and immune cells in pernicious tumor tissues using expression data, was used to acquire immune-related score to predict the infiltration of immune cells in GC. The analytical method is contained in the “estimated” R package (Yan et al., 2019). The abundance of immune cells was quantified through the ssGSEA (Bindea et al., 2013; Finotello and Trajanoski, 2018).

### Function Enrichment Analysis

We sought to identify the signaling pathways by functional enrichment analysis. The R package “LIMMA” was utilized to sort differentially expressed genes (DEGs) between the two ARGs. cluster.The screened DEGs were examined using the R packages “cluster Profiler”, “Rich plot” and “ggplot2” for Gene Ontology (GO), Kyoto Encyclopedia of Genes and Genomes (KEGG) pathway analysis. Gene set enrichment analysis (GSEA) was performed to explore whether the identified sets of genes showed statistical differences between high and low apoptosisScore groups (Subramanian et al., 2005).

### Immunohistochemistry

A total of 50 GC patients’ tissues and corresponding adjacent tissues were collected to explore the expression of six marker genes in the tissue samples by using immunohistochemical staining (IHC). IHC staining was performed according to the manufacturer’s instructions.

### Quantitative Reverse Transcription Polymerase Chain Reaction Assays

Total RNA from tissues was isolated using TRIzol (Invitrogen, Canada) reagent, the specific operation is carried out with reference to the instructions for the operation of the kit. RNA (1 μg) was converted into cDNA using the RevertAid First Strand cDNA Synthesis Kit (Takara, China). qRT-PCR was performed using SYBR Green Mixture (Takara, China) in the ABI StepOne-Plus System (ABI7500, United States). Target gene expression was normalized against GAPDH.

### Statistical Analysis

For the comparison of two groups of continuous variables, the statistical significance of normally distributed variables was estimated by independent Student t-test and differences between non-normally distributed variables were analyzed by Mann-Whitney U-test. Kaplan-Meier survival curves were used to show survival differences, and Log-rank test was used to assess the significance of differences in survival time between two groups of patients. Receiver operating characteristic (ROC) curves were obtained using the pROC R package, and the area under the curve (AUC) was computed to assess the accuracy of the score to estimate prognosis. Heatmap was used by R packages to depict different group information. Additionally, univariate, and multivariate Cox regression analyses were conducted to assess the factors associated with prognosis in GC patients. The analysis was performed using R software of RStudio. Differences were considered significant at *p* < 0.05.

## Results

### Identification of Differential Expression and Prognostic of Apoptosis-Related Genes in TCGA-STAD

**FIGURE 2:**
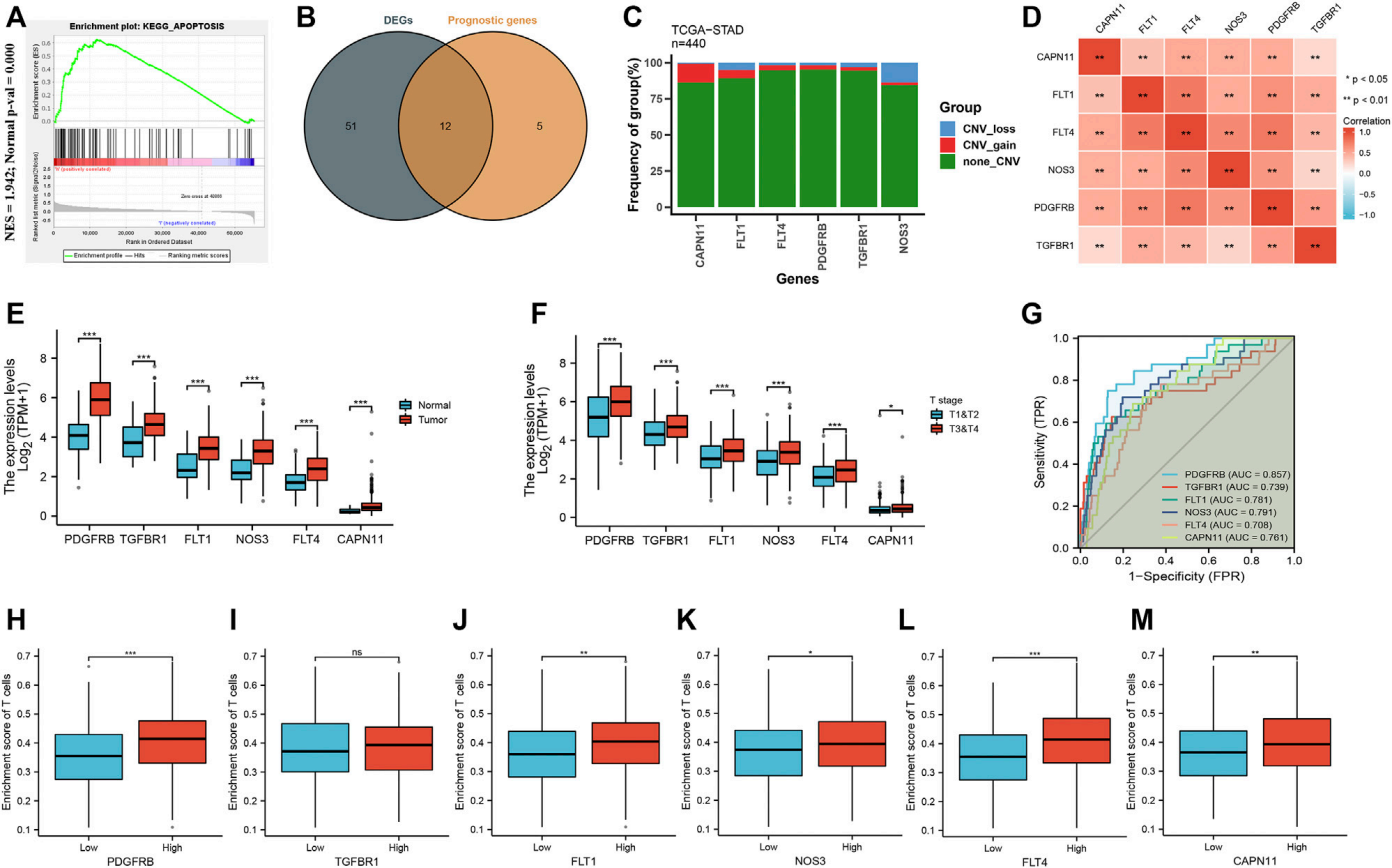
Identification of differential expression and prognostic of ARGs in TCGA-STAD. **(A)** GSEA showed that the apoptosis pathway are differentially enriched in GC patients. **(B)** Venn diagram to identify differentially expressed genes between tumor and normal tissue that were correlated with overall survival. **(C)** Frequencies of CNV gain, loss, and non-CNV among six ARGs. **(D)** Correlation heat map of six ARGs. The size of the colored squares represents the strength of the correlation; red represents a positive correlation. The darker the color is, the stronger correlation is. **(E)** The illustration shows the expression distribution of six ARGs between normal (blue) and GC (red) tissues. **(F)** The illustration shows the expression distribution of six ARGs between T1&T2 (blue) and T3&T4 (red). **(G)** ROC analysis showing that six ARGs has good diagnostic performance. **(H–M)** The correlation between the expression of six ARGs and enrichment score of T cells.

First, GSEA was carried out to determine whether there was a significant apoptosis enrichment between tumor and normal tissues. The results suggested that the apoptosis pathway (Normalize enrichment score = 1.94, adjusted *p*-value = 0.000) was differentially enriched between tumor and normal tissues (Figure 2A). Then, based on the cutoff criterion of |log FC|≥1.0 and adj. *p* < 0.05, 63 different expressions of ARGs were identified (Supplementary Table S2). To explore the prognostic significance of ARGs in GC, univariate Cox regression analysis was performed on ARGs mRNA expression in GC. The analysis revealed that 17 ARGs correlate with OS (Supplementary Table S3). Then, 12 differentially expressed ARGs between tumor and normal tissue that were correlated with OS were identified (Figure 2B). Among them, six ARGs were protective genes, and six ARGs were candidate risky genes. Six risky genes (CAPN11, FLT1, FLT4, NOS3, PDGFRB, TGFBR1) were utilized to form the prognostic signature.

As shown in Figure 2C, we found that five ARGs with a high frequency of CNV gain were highly expressed in GC patients, suggesting that CNVs may be a potential contributor to the regulation of the expression of ARGs. The positive correlation of six ARGs was illustrated in Figure 2D. Furthermore, these ARG expressions were higher in tumor than in normal (Figure 2E), and higher in T3 & T4 than in T1 & T2 (Figure 2F). Moreover, the AUC of ROC for CAPN11, FLT1, FLT4, PDGFRB, TGFBR1, NOS3 was 0.761, 0.781, 0.708, 0.857, 0.739, and 0.791, respectively, suggesting that they have a good diagnostic performance (Figure 2G). As we all know, tumor-infiltrating immune cells are closely connected with the development of cancer. Therefore, we evaluated the correlation between the expression of six ARGs and the enrichment score of T cells. The results showed that higher ARG expression had a higher enrichment score of T cells (Figures 2H–M).

### Molecular Subtype Categorization of Gastric Cancer Based on Six Apoptosis-Related Genes

**FIGURE 3:**
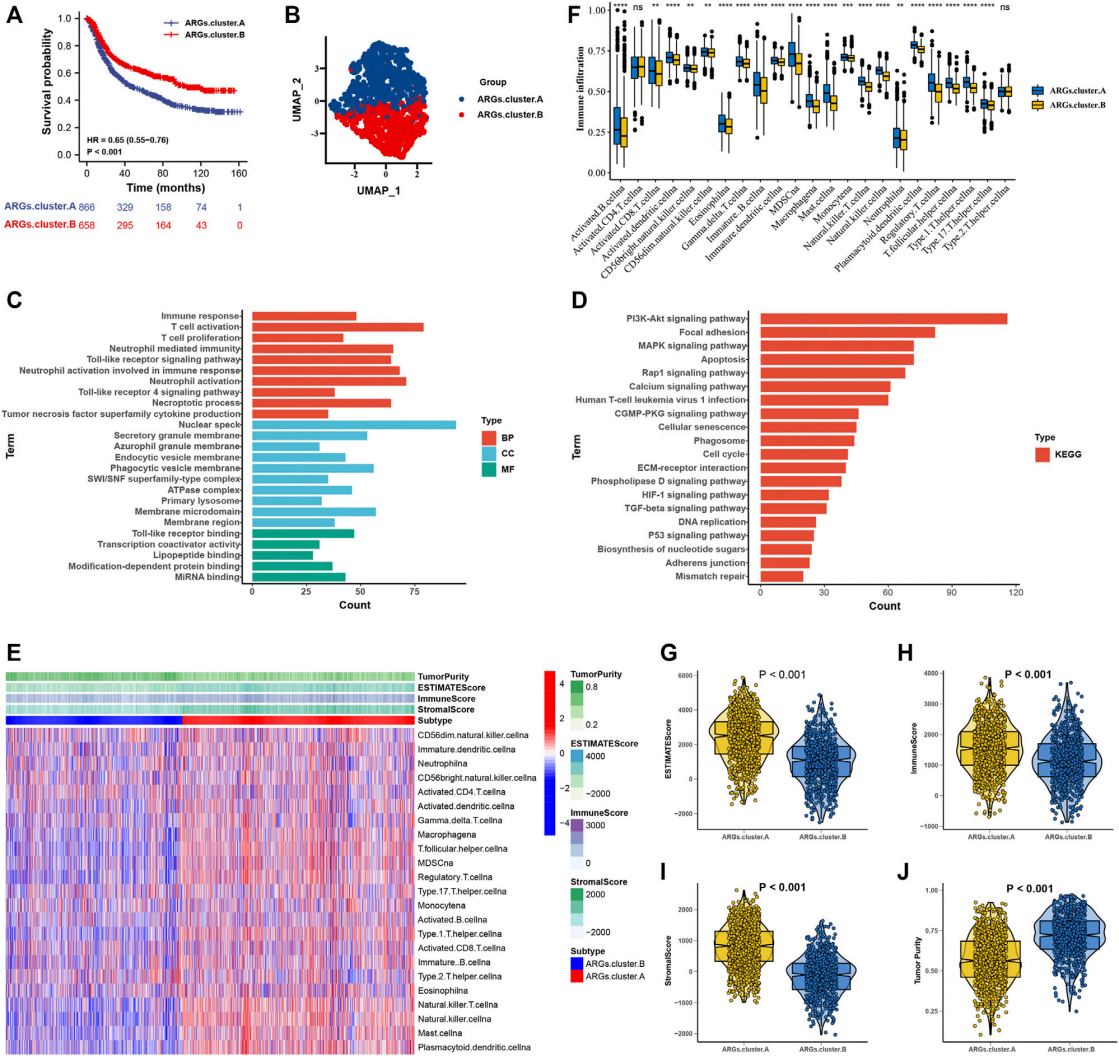
The molecular subtypes categorization of GC base on six ARGs. **(A)** Kaplan-Meier curve showed a significant difference between the two ARGs clusters. **(B)** UMAP analysis for the transcriptome profiles of ARGs.cluster.A and ARGs.cluster.B, showing a remarkable difference on transcriptome between different group. **(C)** GO enrichment analysis **(D)** KEGG enrichment analysis for the different expression that were correlated with OS genes. **(E)** The correlation of tumor microenvironment condition and ARGs.cluster. **(F)** The abundance of each TME infiltrating cell in two ARGs clusters. The upper and lower ends of the boxes represented the interquartile range of values. The lines in the boxes represented median value, and black dots showed outliers. **(G–J)** The ESTIMATE score, stromal score, immune score, and tumor immunity levels in the ARGs.cluster.A and ARGs.cluster.B groups by using ESTIMATE algorithm. (*p < 0.05; **p < 0.01; ***p < 0.001; ****p < 0.0001).

After consensus clustering analysis of these samples by the ConsensusClusterPlus algorithm based on six ARGs, it revealed that the results were most stable at K = 2 (Supplementary Figure S1). Therefore, samples of the GC patients (*N* = 1,524) were divided into ARGs.cluster.A (*N* = 866) and ARGs.cluster.B (*N* = 658) by this approach. Subsequently, based on the grouped results, we collated the clinical information of the correlated OS samples, including the survival status and the overall survival days, the Kaplan-Meier survival analysis was carried out to compare the OS time differences between the different molecular subtypes. It has been shown that patients in the ARGs.cluster.A group have a worse prognosis than the ARGs.cluster.B group (Figure 3A). After obtaining the two molecular subtypes based on consensus cluster analysis, ARGs.cluster.A and ARGs.cluster.B denoted a marked discrimination against each other, suggesting there are DEGs between the two groups (Figure 3B). Finally, 3,083 DEGs that were correlated with OS were identified (Supplementary Table S4). These genes were subjected to GO and KEGG pathway analyses taking advantage of the R package “clusterProfiler, enrichplot, ggplot2”. The genes were chiefly enriched in cell death and immunocyte-related bioprocesses such as necroptotic process, apoptosis, immune response, T-cell activation, T-cell proliferation, neutrophil-mediated immunity, neutrophil activation, and tumor necrosis factor superfamily cytokine production (Figures 3C,D).

The correlation of tumor microenvironment condition and ARGs.cluster was shown in Figure 3E. Using the ssGSEA algorithm, the distribution of immunocyte in patients with GC was plotted in a bar chart, which manifesting that the distribution of various immune cell types varied significantly higher in the ARGs.cluster.A than ARGs.cluster.B (Figure 3F). Using the ESTIMATE algorithm, we discovered that score (ESTIMATE, Stromal, and Immune) was notably higher in the ARGs.cluster.A than ARGs.cluster.B. However, tumor purity score was lower in the ARGs.cluster.A than ARGs.cluster.B (Figures 3G–J). It revealed that group of ARGs.cluster.A patients were helpful for the tumor immunity response. However, effective antitumor immunity was still suppressed.

### Consensus Clustering of 3,083 Genes Identified Two Clusters in Gastric Cancer

**FIGURE 4:**
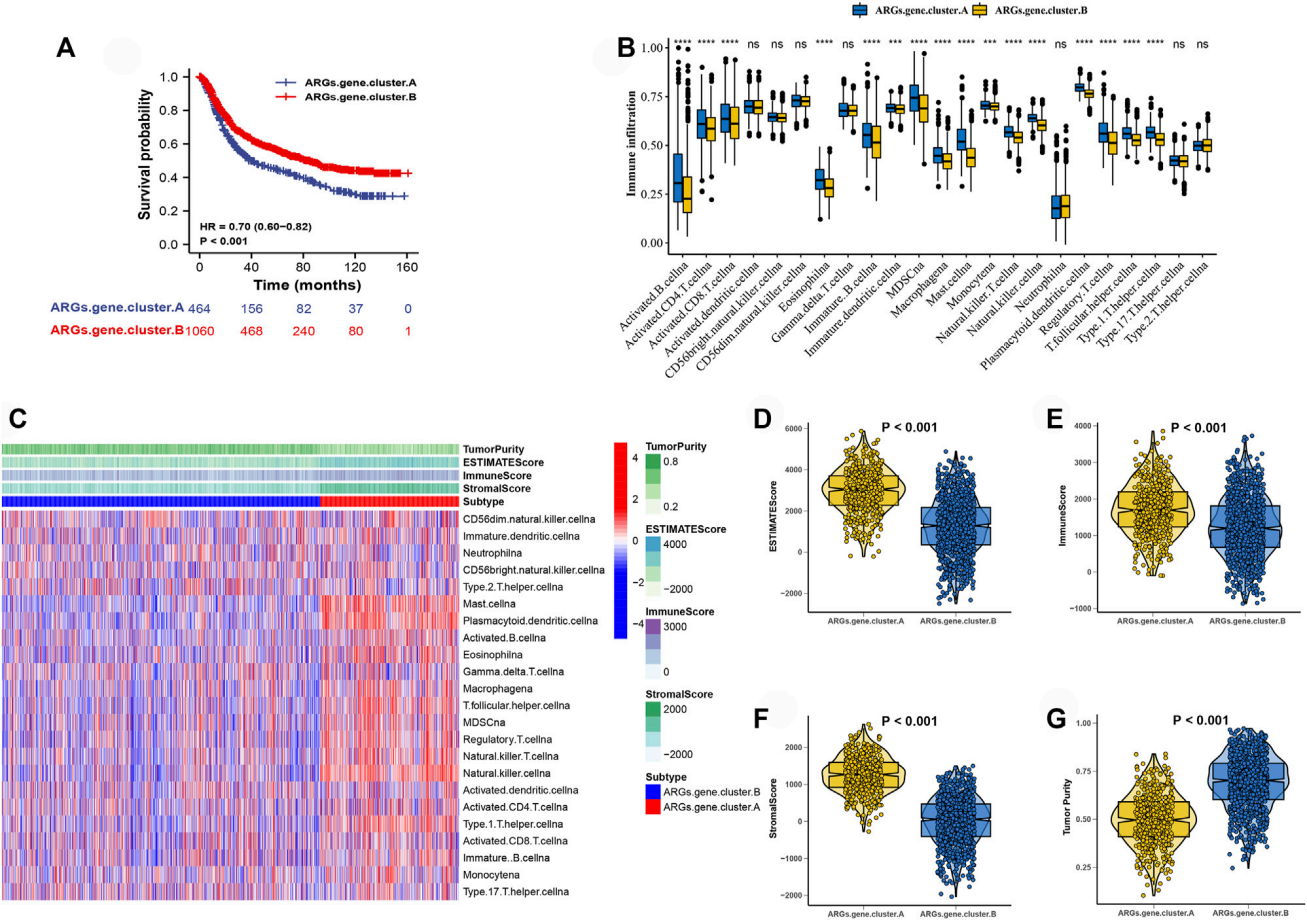
Consensus clustering of 3,083 genes identified two clusters in GC. **(A)** Kaplan-Meier curve showed a significant difference between the two ARGs.gene.clusters. **(B)**The correlation of tumor microenvironment condition and ARGs.gene.clusters. **(C)** The abundance of each TME infiltrating cell in two ARGs.gene.clusters.The upper and lower ends of the boxes represented the interquartile range of values. The lines in the boxes represented median value, and black dots showed outliers. **(D–G)** The ESTIMATE score, stromal score, immune score, and tumor immunity levels in the ARGs.gene.clusters.A and ARGs.gene.clusters.B groups by using ESTIMATE algorithm. (*p < 0.05; **p < 0.01; ***p < 0.001; ****p < 0.0001).

After consensus clustering analysis of these samples by ConsensusClusterPlus algorithm based on 3,083 genes, it revealed that the results were most stable at K = 2 (Supplementary Figure S2). Therefore, samples of the GC patients (*N* = 1,524) were divided into ARGs.gene.cluster.A (*N* = 464) and ARGs.gene.cluster.B (*N* = 1,060) by this approach. Subsequently, based on the grouped results, the Kaplan-Meier survival analysis was carried out to compare the OS time differences between the different molecular subtypes. It has been demonstrated that patients in the ARGs.gene.cluster.A group have a worse prognosis than the ARGs.gene.cluster.B group (Figure 4A).

The correlation of tumor microenvironment condition and ARGs.gene.cluster was shown in Figure 4B. Using the ssGSEA algorithm, the distribution of immunocyte in patients with GC was plotted in a bar chart, which manifesting that the distribution of various immune cell types varied significantly higher in the ARGs.gene.cluster.A than ARGs.gene.cluster.B (Figure 4C). Using the ESTIMATE algorithm, we discovered that score (ESTIMATE, Stromal, and Immune) was notably higher in the ARGs.gene.cluster.A than ARGs.gene.cluster.B. However, tumor purity score was lower in the ARGs.gene.cluster.A than ARGs.gene.cluster.B (Figures 4D–G). It disclosed that group of ARGs.gene.cluster.A patients were helpful for the tumor immunity response. However, the effective antitumor immunity was still suppressed. The above results proved the effectiveness and stability of ARG patterns.

### Establishment of a apoptosisScore

**FIGURE 5:**
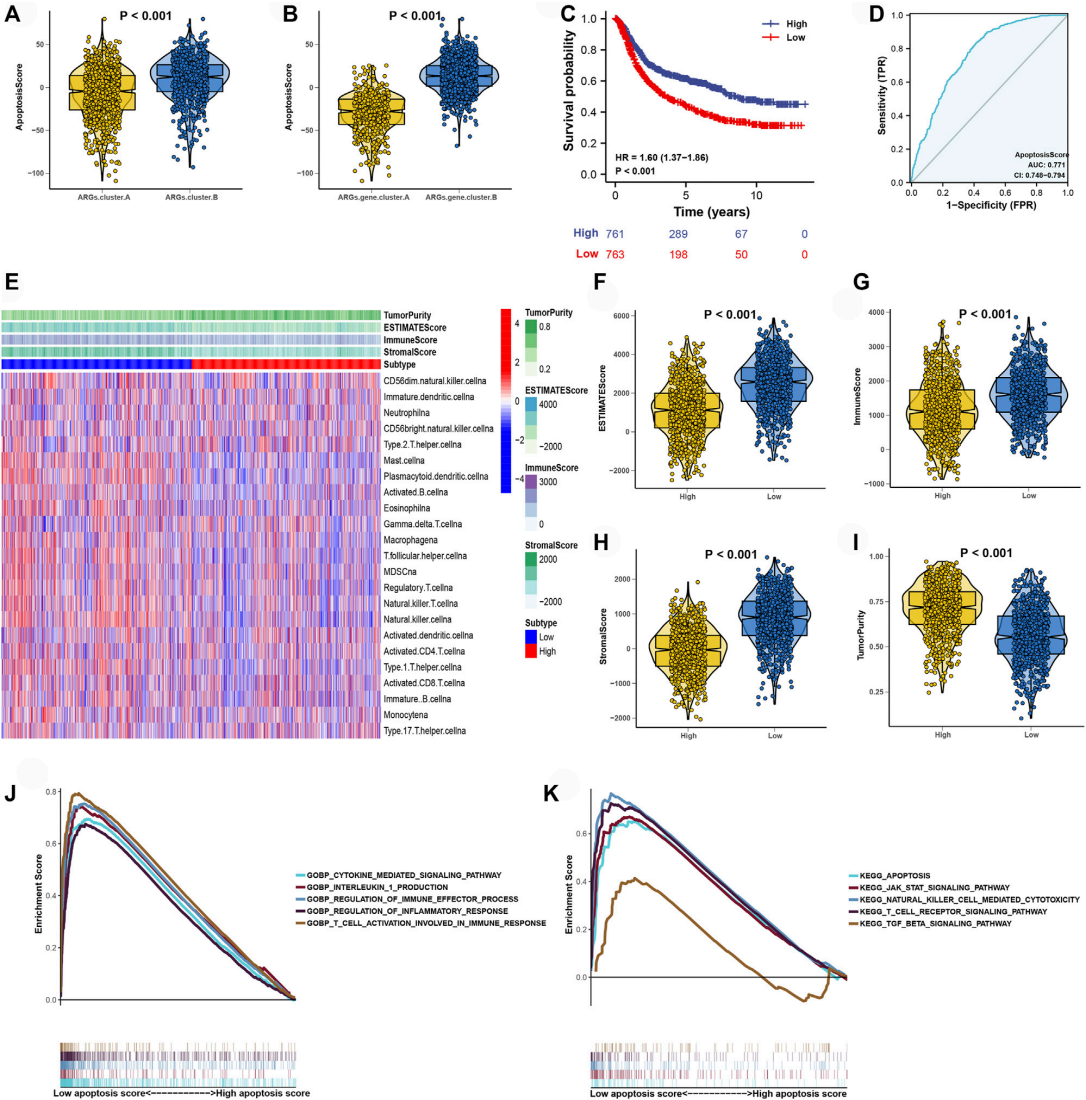
Construction of apoptosisScore. **(A)** Differences in apoptosisScore among two ARGs. **(B)** Differences in apoptosisScore among two ARGs.gene.clusters. **(C)** Kaplan-Meier curves for high and low apoptosisScore groups. **(D)** The predictive value of apoptosisScore. **(E)** The correlation of tumor microenvironment condition in high and low apoptosisScore patient groups. **(F–I)** The ESTIMATE score, stromal score, immune score, and tumor immunity levels in high and low apoptosisScore groups by using ESTIMATE algorithm. **(J)** GSEA GO identified high and low apoptosisScore groups related signaling pathways in GC. **(K)** GSEA KEGG identified high and low apoptosisScore related signaling pathways in GC.

We constructed an apoptosisScore base on 3,083 genes to quantify the apoptosis index of each GC patient by using the PCA method (Supplementary Table S5). We discovered that apoptosisScore was notably lower in ARGs.cluster.A and ARGs.gene.cluster.A than in ARGs.cluster.B and ARGs.gene.cluster.B (Figures 5A,B). Meanwhile, it has been shown that patients with the low apoptosisScore had a worse prognosis than the high apoptosisScore (Figure 5C). Moreover, the AUC of ROC for apoptosisScore was 0.77, suggesting that they have a superior diagnostic performance (Figure 5D). The correlation between tumor microenvironment condition and apoptosisScore was illustrated in Figure 5E. It indicated that the relative abundance of most of the infiltrating immune cell types increased in the low apoptosisScore group than in high apoptosisScore group (Supplementary Figure S3). We also discovered that score (ESTIMATE, Stromal, and Immune) was notably higher in low apoptosisScore group than the high apoptosisScore group. However, the tumor purity score was lower in the low apoptosisScore group than the high apoptosisScore group (Figures 5–I). These results indicate that tumor-infiltrating lymphocytes in the low apoptosisScore group were unable to recruit to the tumor site.

Then, the GSEA results suggested that regulation of the immune effector process, regulation of inflammatory response, T-cell activation involved in immune response, apoptosis pathway, natural killer cell-mediated cytotoxcity pathway, and T-cell receptor signaling pathway were differentially enriched in low apoptosisScore phenotypes (Figures 5J,K). Function enrichment analysis results strongly indicated that low apoptosisScore is closely related to the tumor immune microenvironment (TIME), and thus worthy of further analysis.

### Prognostic Value of apoptosisScore in Gastric Cancer Patients

**FIGURE 6:**
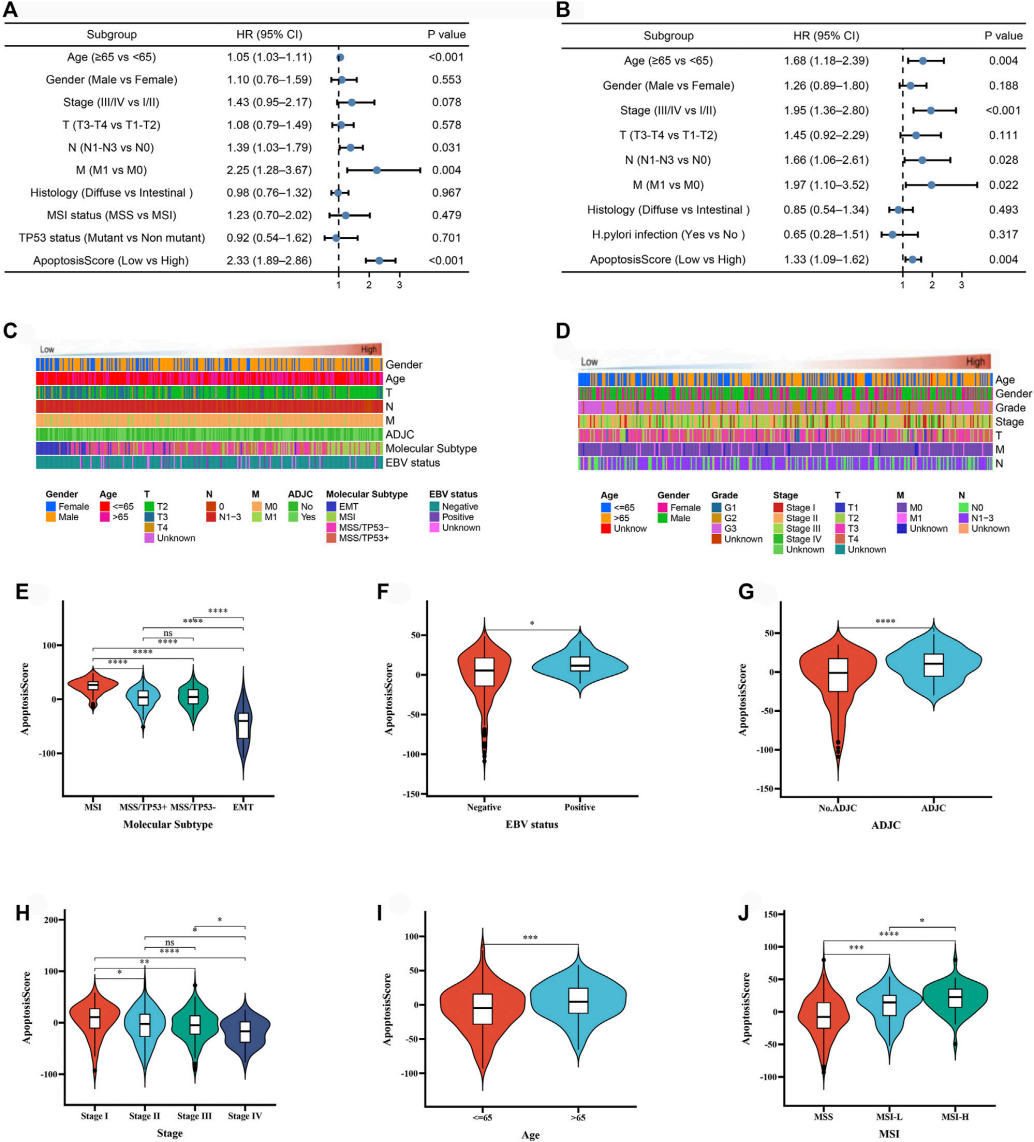
The prognostic value of apoptosisScore in GC patients. **(A)** Multivariate Cox regression analysis for apoptosisScore in ACRG cohort shown by the forest plot. **(B)** Multivariate Cox regression analysis for apoptosisScore in TCGA cohort shown by the forest plot. **(C)** Heatmap showing the dependence between apoptosisScore and clinicopathologic characteristics in ACRG cohort. **(D)** Heatmap showing the dependence between apoptosisScore and clinicopathologic characteristics in TCGA cohort. **(E–G)** Differences in apoptosisScore between molecular subtypes, EBV status, ADJC status in ACRG cohort. **(H–J)** Differences in apoptosisScore between stage, age, MSI status in TCGA cohort.

In the above, we reported that apoptosisScore may have prognostic significance in GC. In order to further explore apoptosisScore as a separate prognostic indicator in GC patients. Multivariate analyses showed that low apoptosisScore was a separate prognostic indicator in GC patients (ACRG cohort: HR = 2.33, 95% CI = 1.89–2.86, *p* < 0.001, TCGA cohort: HR = 1.33, 95% CI = 1.09–1.62, *p* = 0.004) (Figures 6A,B).

To continue exploring the potential malignant behavior of apoptosisScore, patients with apoptosisScore in the TCGA and ACRG cohorts were analyzed (Figures 6C,D). Specifically, we compared the differential level of apoptosisScore in different subgroups stratified by molecular subtypes, EBV status, ADJC status in ACRG cohort, stage, age, MSI status in TCGA cohort. As shown in Figures 6E–J, based on the ACRG cohort, apoptosisScore was significantly different among subgroups stratified by molecular subtypes, EBV status, ADJC status, in ACRG cohort, and stage, age, MSI status in TCGA cohort.

### Internal and External Validation of apoptosisScore

**FIGURE 7:**
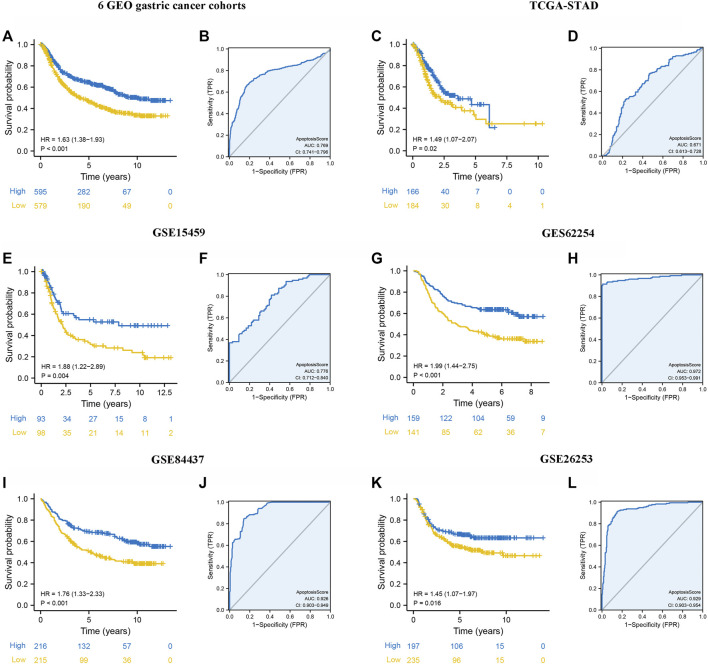
Internal and external validation of apoptosisScore. **(A)** Kaplan-Meier curves for high and low apoptosisScore groups in six GEO datasets (GSE84437, GSE34942, GSE15459, GSE57303, ACRG/GSE62254, and GSE29272). **(B)** The predictive value of apoptosisScore in six GEO dataset. **(C)** Kaplan-Meier curves for high and low apoptosisScore groups in TCGA-STAD. **(D)** The predictive value of apoptosisScore in TCGA-STAD. **(E)** Kaplan-Meier curves for high and low apoptosisScore groups in GSE15459. **(F)** The predictive value of apoptosisScore in GSE15459. **(G)** Kaplan-Meier curves for high and low apoptosisScore groups in GSE62254. **(H)** The predictive value of apoptosisScore in GSE62254. **(I)** Kaplan-Meier curves for high and low apoptosisScore patient groups in GSE84437. **(J)** The predictive value of apoptosisScore in GSE84437. **(K)** Relapse-free survival analysis of apoptosisScore in GSE26253 cohort. **(L)** The predictive value of apoptosisScore in GSE26253.

Kaplan-Meier survival analysis was conducted to contrast the OS of patients with high and low apoptosisScore and determine whether apoptosisScore can be used as a stable marker for GC patients. The similar above results showed that patients with low apoptosisScore had poorer OS (6 GEO cohorts: HR = 1.63, 95% CI = 1.38–1.93, *p* < 0.001, TCGA-STAD cohort: HR = 1.49, 95% CI = 1.07–2.07, *p* = 0.020, GSE15459 cohort: HR = 1.88, 95% CI = 1.22–2.89, *p* = 0.004, GSE62245 cohort: HR = 1.99, 95% CI = 1.44–2.75, *p* < 0.001, GSE84437 cohort: HR = 1.76, 95% CI = 1.33–2.33, *p* = 0.020) (Figures 6A,C,E,G,I). The AUC of ROC for apoptosisScore were 0.769 in six GEO cohorts, 0.671 in TCGA-STAD cohort, 0.776 in GSE15459 cohort, 0.972 in GSE62245 cohort, 0.926 in GSE84437 cohort, respectively, suggesting that a prominent fitting prediction (Figures 7B,D,E,H,J). In addition, the results also showed that patients with low apoptosisScore had poorer RFS (GSE26253 cohort: HR = 1.45, 95% CI = 1.07–1.97, *p* = 0.016) (Figure 7K). The AUC of ROC for apoptosisScore was 0.829 in GSE26253 cohort, also had a prominent fitting prediction in GSE26253 cohort (Figure 7L).

### Prognostic Validation of apoptosisScore in the Immune IMvigor210 Cohort

**FIGURE 8:**
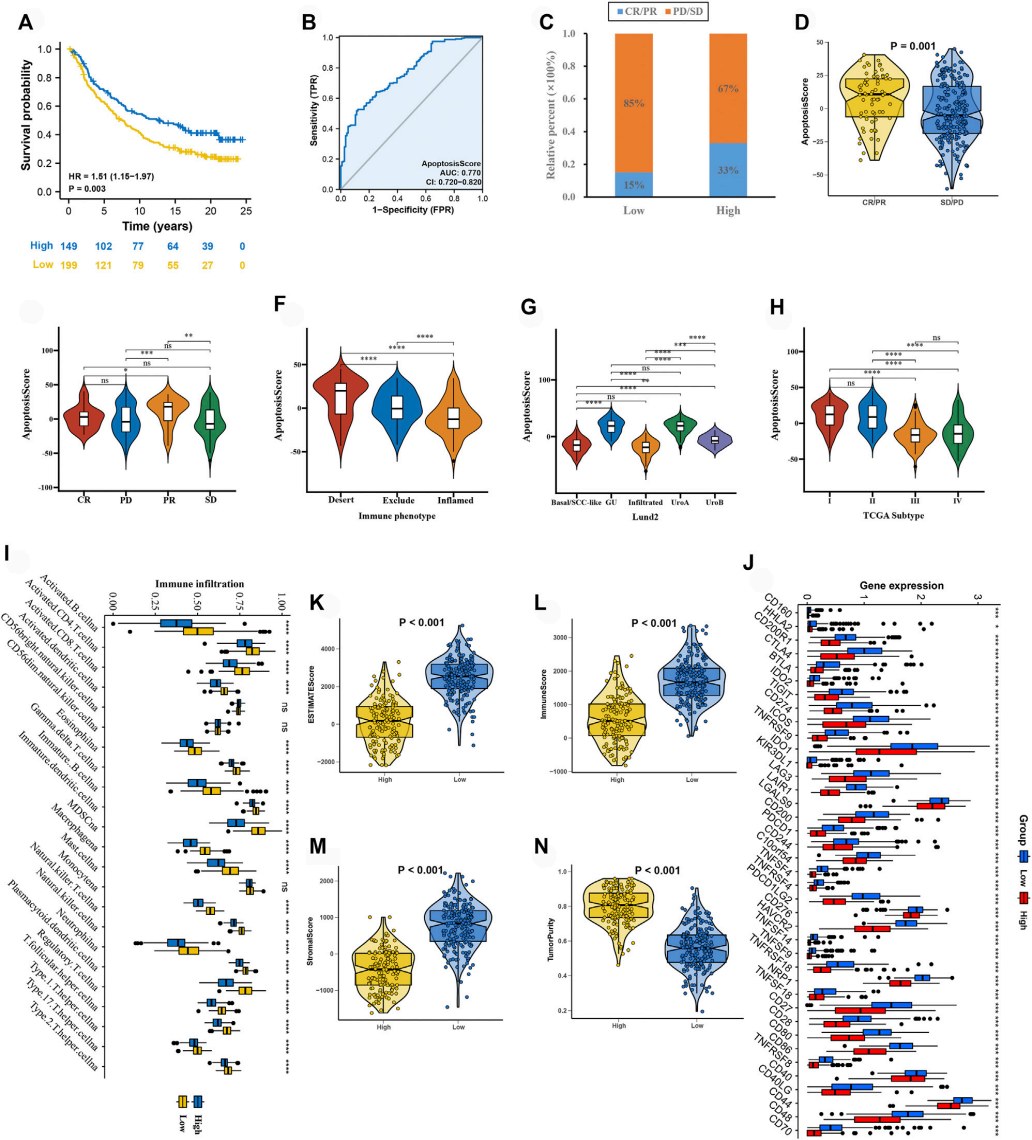
ApoptosisScore in the role of anti-PD-1/L1 immunotherapy in IMvigor210 cohort. **(A)** Survival analyses for low and high apoptosisScore groups in IMvigor210 cohort. **(B)** The predictive value of apoptosisScore in IMvigor210 cohort. **(C)** The proportion of patients with response to PD-L1 blockade immunotherapy in low or high apoptosisScore groups. **(D)** Differences in apoptosisScore among distinct anti-PD-1 clinical response groups. **(E)** Distribution of apoptosisScore in distinct anti-PD-L1 clinical response groups. **(F–H)** Differences in apoptosisScore between immune subtypes, Lund2 subtypes, and TCGA subtypes. **(I)** The abundance of each TME infiltrating cell in high and low apoptosisScore groups. **(J)** Differences in checkpoint expression between low and high apoptosisScore groups. **(K–N)** The ESTIMATE score, stromal score, immune score, and tumor immunity levels in high and low apoptosisScore groups by using ESTIMATE algorithm.

First, after consensus clustering analysis of these samples by ConsensusClusterPlus algorithm based on six ARGs in IMvigor210 cohort, it revealed that the results were most stable at K = 2 (Supplementary Figure S4). Then, 1822 DEGs that were correlated with OS were identified (Supplementary Table S6). Finally, we constructed an apoptosisScore base on 1822 genes (Supplementary Table S7). Based on the IMvigor210 cohort, we performed survival analyses, patients with low apoptosisScore had a worse clinical prognosis for bladder cancer (HR = 1.51, 95% CI = 1.15–1.97, *p* = 0.003) (Figure 8A). The AUC of ROC for apoptosisScore was 0.770 in IMvigor210 cohort, and also had a prominent fitting prediction in IMvigor210 cohort (Figure 8B). The proportions of complete response (CR)/partial response (PR) and stable disease (SD)/progressive disease (PD) were 15 and 85% in the low apoptosisScore group and 33 and 67% in the high apoptosisScore group, correspondingly (*p* < 0.05) (Figure 8C). ApoptosisScore also varied statistically in the CR/PR and SD/PD groups, apoptosisScore was notably lower in SD/PD group than CR/PR group (Figure 8D). We also found that apoptosisScore was notably lower in SD or PD groups than in CR or PR groups (Figure 8E). The above results suggest that apoptosisScore was sensitive to immunotherapy. Various histologically and transcriptionally immune tumor subtypes were distinguished, including inflamed, excluded, and desert immune tumors. Various histologically and transcriptionally immune tumor subtypes were distinguished, including inflamed, excluded, and desert immune tumors. ApoptosisScore also varied statistically in the three immune tumor subtypes, five Lund2 subtypes, and three TCGA subtypes, suggesting that apoptosisScore is closely related to the proposed immune subtypes and molecular subtypes (Figures 8F–H) (Table 2).

**TABLE 2:** Summary of detailed clinical information of IMvigor210 (mUC) cohort.

IMvigor210 Cohort	Low (*n* = 199)	High (*n* = 149)	Total (*n* = 348)
Vital Status			
Alive	54 (27.1%)	62 (41.6%)	116 (33.3%)
Dead	145 (72.9%)	87 (58.4%)	232 (66.7%)
**Gender**			
Female	41 (20.6%)	35 (23.5%)	76 (21.8%)
Male	158 (79.4%)	114 (76.5%)	272 (78.2%)
**Overall response**			
CR	14 (8.2%)	11 (8.7%)	25 (8.4%)
PR	12 (7.0%)	31 (24.4%)	43 (14.4%)
SD	40 (23.4%)	23 (18.1%)	63 (21.1%)
PD	105 (61.4%)	62 (48.8%)	167 (56.0%)
**Binary response**			
CR/PR	26 (15.2%)	42 (33.1%)	68 (22.8%)
SD/PD	145 (84.8%)	85 (66.9%)	230 (77.2%)
**Enrollment IC**			
IC0	41 (20.6%)	58 (38.9%)	99 (28.4%)
IC1	75 (37.7%)	57 (38.3%)	132 (37.9%)
IC2	83 (41.7%)	34 (22.8%)	117 (33.6%)
**IC level**			
IC0	38 (19.2%)	59 (39.6%)	97 (28.0%)
IC1	76 (38.4%)	56 (37.6%)	132 (38.0%)
IC2+	84 (42.4%)	34 (22.8%)	118 (34.0%)
**TC Level**			
TC0	145 (73.2%)	130 (87.2%)	275 (79.3%)
TC1	10 (5.1%)	12 (8.1%)	22 (6.3%)
TC2+	43 (21.7%)	7 (4.7%)	50 (14.4%)
**Immune phenotype**			
Desert	27 (16.9%)	49 (39.5%)	76 (26.8%)
Excluded	75 (46.9%)	59 (47.6%)	134 (47.2%)
Inflamed	58 (36.3%)	16 (12.9%)	74 (26.1%)
**TCGA cluster**			
I	44 (22.1%)	74 (49.7%)	118 (33.9%)
II	41 (20.6%)	54 (36.2%)	95 (27.3%)
III	61 (30.7%)	8 (5.4%)	69 (19.8%)
IV	53 (26.6%)	13 (8.7%)	66 (19.0%)
**Lund2**			
Basal/SCC-like	62 (31.2%)	4 (2.7%)	66 (19.0%)
Genomically unstable	14 (7.0%)	56 (37.6%)	70 (20.1%)
Infiltrated	90 (45.2%)	2 (1.3%)	92 (26.4%)
UroA	17 (8.5%)	85 (57.0%)	102 (29.3%)
UroB	16 (8.0%)	2 (1.3%)	18 (5.2%)

Using the ssGSEA algorithm, the distribution of immunocyte in patients with GC was plotted in a bar chart, which manifests that the distribution of various immune cell types varied significantly higher in the low apoptosisScore than in high apoptosisScore (Figure 8I). Utilizing the ESTIMATE algorithm, we discovered that score (ESTIMATE, Stromal, and Immune) was notably higher in the low apoptosisScore group than high apoptosisScore group. However, the tumor purity score was lower in the low apoptosisScore group than high apoptosisScore group (Figures 8K–N). It revealed that the group of low apoptosisScore group patients was helpful for the tumor immunity response. However, effective antitumor immunity was still suppressed. Based on patients with bladder cancer in the IMvigor210 cohort, the relationship between apoptosisScore and immune checkpoint genes was investigated. We found that immune checkpoint genes were significantly higher in the low apoptosisScore group than in high apoptosisScore group, which was consistent with the “immunity tidal model theory” (Figure 8J).

**FIGURE 9:**
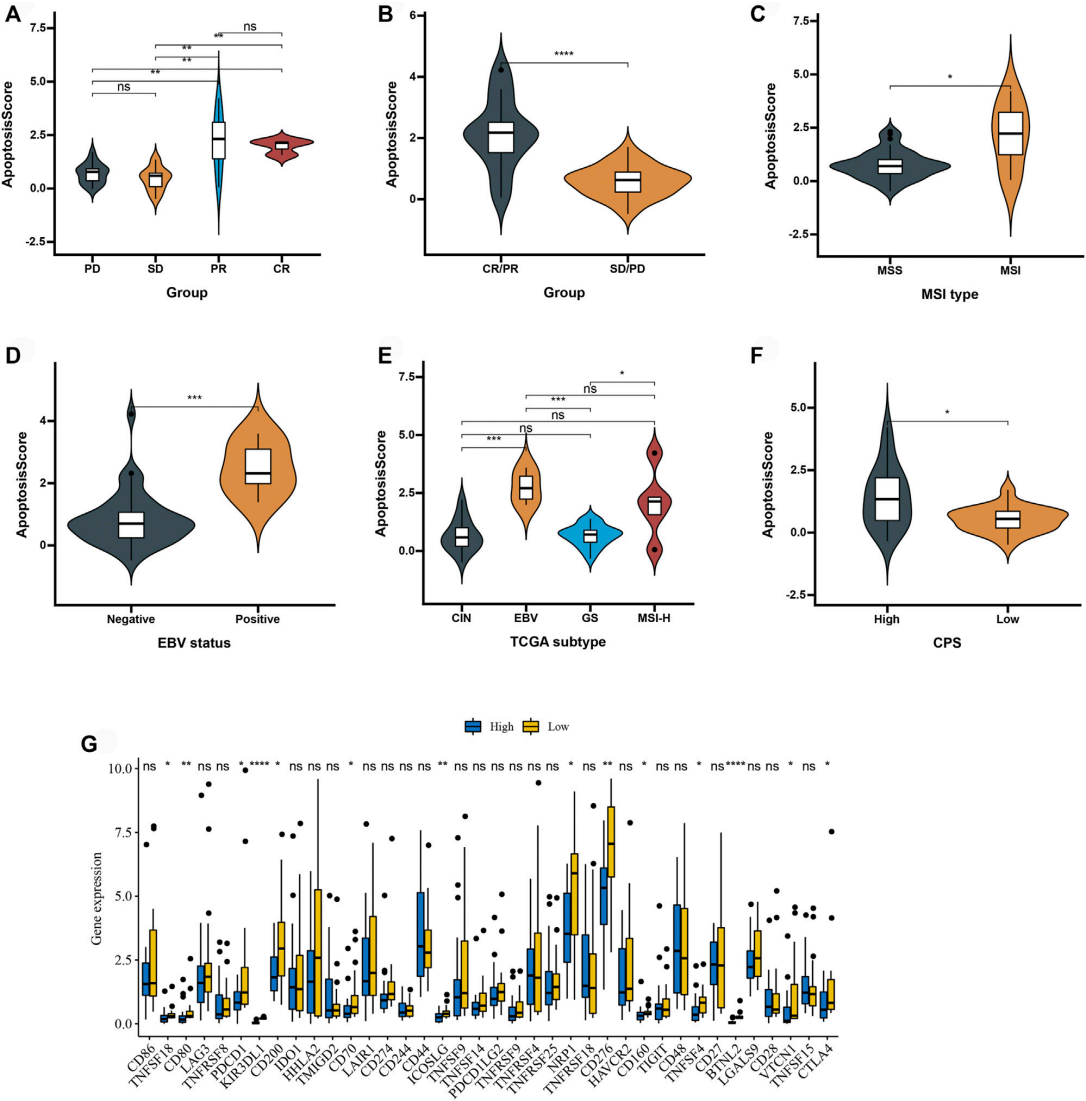
Patient characteristics and apoptosisScore of advanced gastric cancer treated with anti-PD-1 immmunotherapy. **(A)** Distribution of apoptosisScore in distinct anti-PD-L1 clinical response groups. **(B)** Differences in apoptosisScore among distinct anti-PD-1 clinical response groups. **(C)** Differences in apoptosisScore between MSI type. **(D)** Differences in apoptosisScore between EBV status. **(E)** Differences in apoptosisScore between TCGA subtypes. **(F)** Differences in apoptosisScore between high and low CPS score groups. **(G)** Differences in checkpoint expression between low and high apoptosisScore groups in advanced gastric cancer treated with anti-PD-1 immmunotherapy.

Next, we used an advanced gastric cancer immune cohort (Kim cohort) to detect the relationship between apoptosisScore and immunotherapy. The detail of patient characteristics and apoptosisScore of advanced gastric cancer treated with anti-PD-1 immunotherapy was shown in Supplementary Table S8. Specifically, apoptosisScore also varied statistically in the CR, PR, SD, and PD groups, apoptosisScore was notably lower in SD or PD groups than in CR or PR groups (Figure 9A). We also discovered that apoptosisScore was notably lower in SD/PD group than in CR/PR group (Figure 9B). The above results suggest that apoptosisScore was sensitive to immunotherapy. We also compared the differential level of apoptosisScore in different subgroups stratified by MSI type, EBV status, TCGA subtypes, and CPS score groups. As shown in Figures 9C–F, based on the Kim cohort, apoptosisScore was significantly higher in MSI type group, positive EBV status group, EBV group, MSI-H group and high CPS score group. We found that immune checkpoint genes were significantly higher in the low apoptosisScore group than in high apoptosisScore group (Figure 9G).

### Validation the Prognosis of apoptosisScore in Gastric Cancer in an Independent Cohort

**FIGURE 10:**
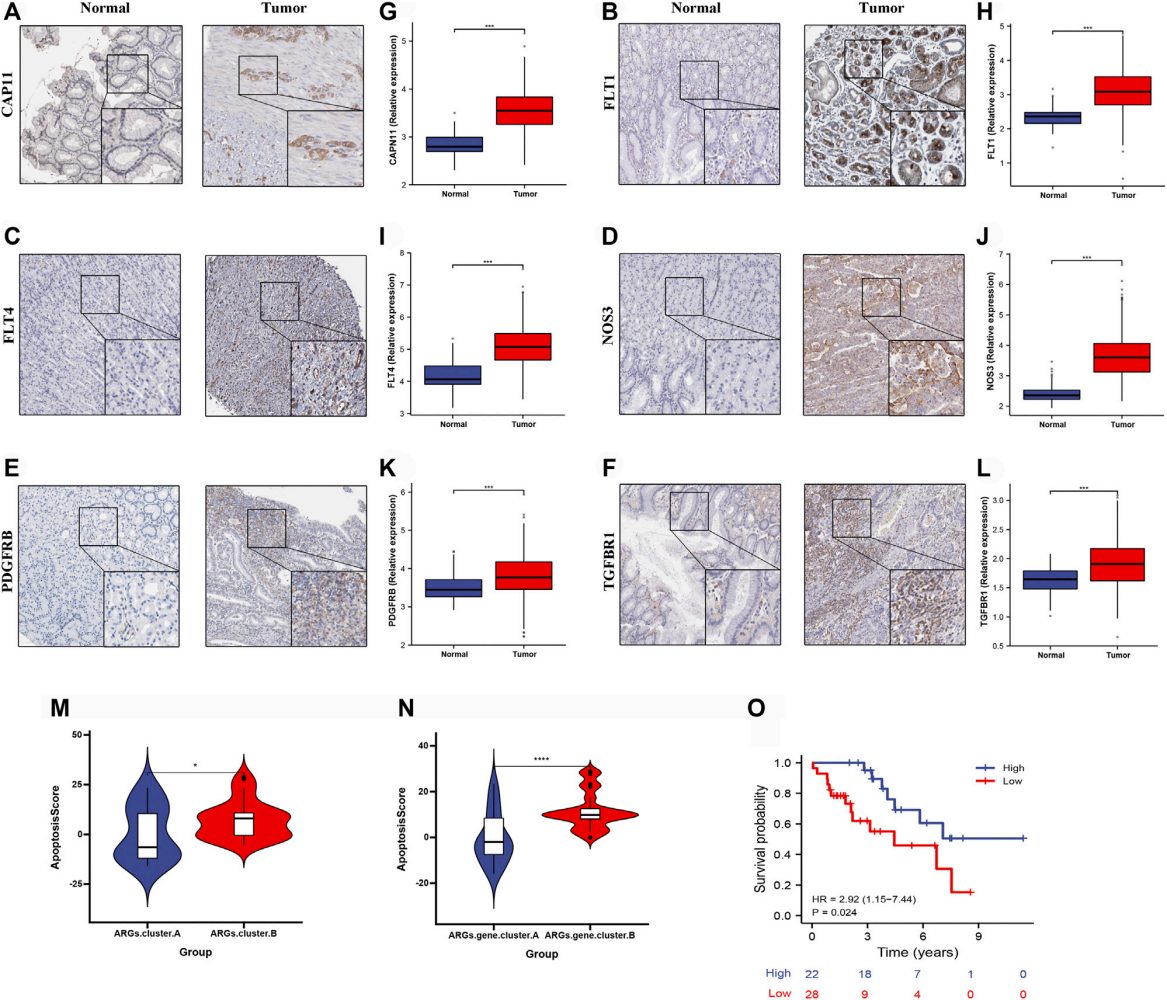
The expression and overall survival of six ARGs in GC. **(A–F)** Representative immunohistochemistry images of six ARGs expression in normal tissues, and GC tissue. **(G–L)** six ARGs mRNA levels are shown for the GC and normal tissue. **(M)** Differences in apoptosisScore among two ARGs. **(N)** Differences in apoptosisScore among two ARGs.gene.clusters. **(O)** Kaplan-Meier analysis of overall survival based on apoptosisScore in 50 cases of GC patients.

To further validate apoptosisScore in GC, we measured the six ARG protein levels by immunohistochemistry, and the result showed that compared with normal group, the CAPN11, FLT1, FLT4, NOS3, PDGFRB, and TGFBR1 levels were significantly higher in GC group (Figures 10A–F). In addition, RT-qPCR was used to detect the six marker genes mRNA expression in GC. Compared with the normal group, the six marker genes mRNA level was significantly higher in the GC group (Figures 10G–L). We also discovered that apoptosisScore was notably lower in ARGs.cluster.A and ARGs.gene.cluster.A than in ARGs.cluster.B and ARGs.gene.cluster.B (Figures 10M,N). Kaplan-Meier analysis revealed that the prognosis of patients with low apoptosisScore was significantly poor than that of patients with high apoptosisScore (Figure 10O).

## Discussion

In this study, analysis of information from the eight GC cohorts and immune IMvigor210 cohort indicated that low apoptosisScore is related to poor prognosis for GC. We investigated that low apoptosisScore was interrelated with clinicopathologic features such as molecular subtypes, EBV status, ADJC status, stage, age, and MSI status, suggesting that low apoptosisScore participates in a role in the malignant behavior of GC. Multivariate Cox regression analyses indicated that low apoptosisScore is an independent adverse factor affecting the prognosis of GC patients, which was also confirmed in five additional validated cohorts. Kaplan-Meier survival analysis was utilized to contrast the OS of patients with high and low apoptosisScore, and the results showed that low apoptosisScore could be used as a prognostic indicator for GC patients. ROC curve evaluation suggested that apoptosisScore could be invoked as a useful diagnostic marker. Moreover, apoptosisScore may also participate in a critical part in TIME of GC by regulating the infiltration of immune cells, suggesting that apoptosisScore might be used as a therapeutic target to regulate the anti-tumor immune response.

We discovered that 3,083 DEGs were correlated with OS between the two ARGs.cluster, which is correlated with various immune-related biological processes. Functional enrichment analysis found that these genes in GC patients, which has a significant impact on immune-related biological processes, primarily manifests in immune response, T-cell activation, T-cell proliferation, neutrophil-mediated immunity, Toll-like receptors (TLRs), HIF-1 signaling pathway, P53 signaling pathway, and neutrophil activation. GSEA findings indicated that a low apoptosisScore phenotype is positively correlated with processes related to immune effector process, regulation of inflammatory response, T-cell activation involved in immune response, natural killer cell-mediated cytotoxcity pathway, and T-cell receptor signaling pathway. Moreover, TLRs belong to the pattern recognition receptor superfamily, which typically activates and mediate the pro-inflammatory response of innate immune cells by identifying invading pathogens (Vinnakota et al., 2013). Hypoxia can lead to maladjustment of cell cycle checkpoint by inducing posttranslational modification of wild-type p53, ultimately promoting malignant tumor progression (Cobbs et al., 2003). In particular, antigen-targeting cytotoxicity of T lymphocytes is now identified as a critical factor in the relationship between the immune system and cancer prevention (Waldman et al., 2020). Interestingly, our study indicates that low apoptosisScore is closely interrelated with the above-mentioned immune pathways, and therefore, we believe that low apoptosisScore is closely interrelated with the TIME of GC tissues.

In essence, we identified an interrelation between ARGs.cluster, apoptosisScore, and TIME, in which the behavior of tumor cells determines the outcome of the tumor and affects the biology of TIME cells (Ansell and Vonderheide, 2013). Using the ESTIMATE and ssGSEA algorithm, we discovered that infiltrating immune cell types, ESTIMATE score, stromal score, and the immune score increased in the low apoptosisScore group, and tumor purity decreased in the low apoptosisScore group. The immune score was originally used to assess the stage and prognosis of cancer patients, and patients with a high immune score generally have a better prognosis (Galon et al., 2012). However, Kaplan-Meier survival analysis in the present study found that the clinical outcome of patients with high low apoptosisScore was markedly worse than that of the high apoptosisScore group. Therefore, we hypothesize that apoptosisScore affects the type of immune infiltrating cells in GC. TFHs aid the activity of B cells in germinal center responses and reduce immunosuppression through the inflammatory response and helping to organize tertiary lymphoid structures to achieve anti-tumor effects, which was reported in breast, colorectal and other tumors (Gu-Trantien et al., 2013; Hetta et al., 2020). We found that immune checkpoint genes were significantly higher in low apoptosisScore group than in high apoptosisScore group, which was consistent with the “immunity tidal model theory” that high expression of both conciliatory and contributory immune checkpoints caused an immunosuppressive phenotype in tumors (Zhu et al., 2011). However, the precise mechanism requires further study.

The development of new immunotherapies has advanced rapidly in the field of oncology in recent years, and immune checkpoint inhibition is examined as a potentially important method for the treatment of GC. Mechanisms that suppress the activation and/or effector function of immune cells are called immune checkpoints (Kalbasi and Ribas, 2020; Wang et al., 2020). In this study, we examined the dependence between apoptosisScore and various immune checkpoints. Our result showed that immune checkpoint genes were significantly higher in low apoptosisScore group than in high apoptosisScore group. Indeed, considerable progress has been made in targeting these receptors, such as PDCD1/PDL1 (also known as CD274), CD47 blockade therapies (Barclay and Van den Berg, 2014; Feng et al., 2019). A recent tumor study found that TGFBR1 modified T-cell function by blocking the PD-1/PD-L1 checkpoint, to achieve an anti-tumor effect (Neviani et al., 2019). These studies show that apoptosis participates as a critical part in the PD-1/PD-L1 axis, similar to our results in that low apoptosisScore was positively correlated with PD-1 and PD-L1. It is worth noting that the first generation of immune checkpoint inhibitors, such as inhibitors of CTLA-4 and PD-1, targeted the most distinctive immune checkpoints and thus represents the most mature immunotherapy agents (Marshall and Djamgoz, 2018). Moreover, low apoptosisScore was correlated with other second-generation immune checkpoint genes, including CD40, TNFSF14, BTLA, and HAVCR2. Thus, we considered the possibility of low apoptosisScore as a novel therapeutic target for GC patients, as it could provide an important new basis and direction for immunotherapy in GC patients. As tumor immune resistance is characterized by the co-expression of multiple immune checkpoint pathway molecules, double or multiple checkpoints blocked may produce more powerful anti-tumor immunotherapy effects; therefore, additional immune targets need to be identified.

There are numerous restrictions in the present study. First, this study was inspired by preliminary data and hypothesis-generating predictions. Second, although we found that apoptosisScore was interrelated with patient prognosis and immune invasion in GC, we could not prove that low apoptosisScore affects prognosis through immune invasion, which has to be verified using cell lines. In addition, elucidating the mechanisms by which apoptosisScore regulates the infiltration of immune cells will require further study. The experimental evidence of whether the expression of apoptosisScore will affect the abundance of immune cell infiltration is a deficiency of our study, and it will become a direction of follow-up research. The apoptosisScore with an AUC of 0.671 in TCGA-STAD cohort, which is lower than PDGFRB, FLT1, NOS3, and higher than CAPN11, FLT4, TGFBR1. However, the AUC of apoptosisScore is 0.972 in ACRG (Asian Cancer Research Group)/GSE62254. Therefore, the prediction accuracy of apoptosisScore is higher among Asian people, the results of our study should be further validated using more multicenter clinical data. However, this subject is new and worthy of further study.

In conclusion, our study suggests that low apoptosisScore is a latent marker for determining the prognosis of GC patients. Low apoptosisScore may also participate a critical part in TIME of GC by egulating the infiltration of immune cells, suggesting that low apoptosisScore might be used as a therapeutic target to regulate the anti-tumor immune response.

## Data Availability

The original contributions presented in the study are included in the article/Supplementary Material; further inquiries can be directed to the corresponding author.
