# Supplementary figures and images for: Apoptosis-Related Gene-Mediated Cell Death Pattern Induces Immunosuppression and Immunotherapy Resistance in Gastric Cancer
Source: Front Genet. 2022 Jul 5;13:921163. doi: 10.3389/fgene.2022.921163 (PMC9295743; doi:10.3389/fgene.2022.921163)

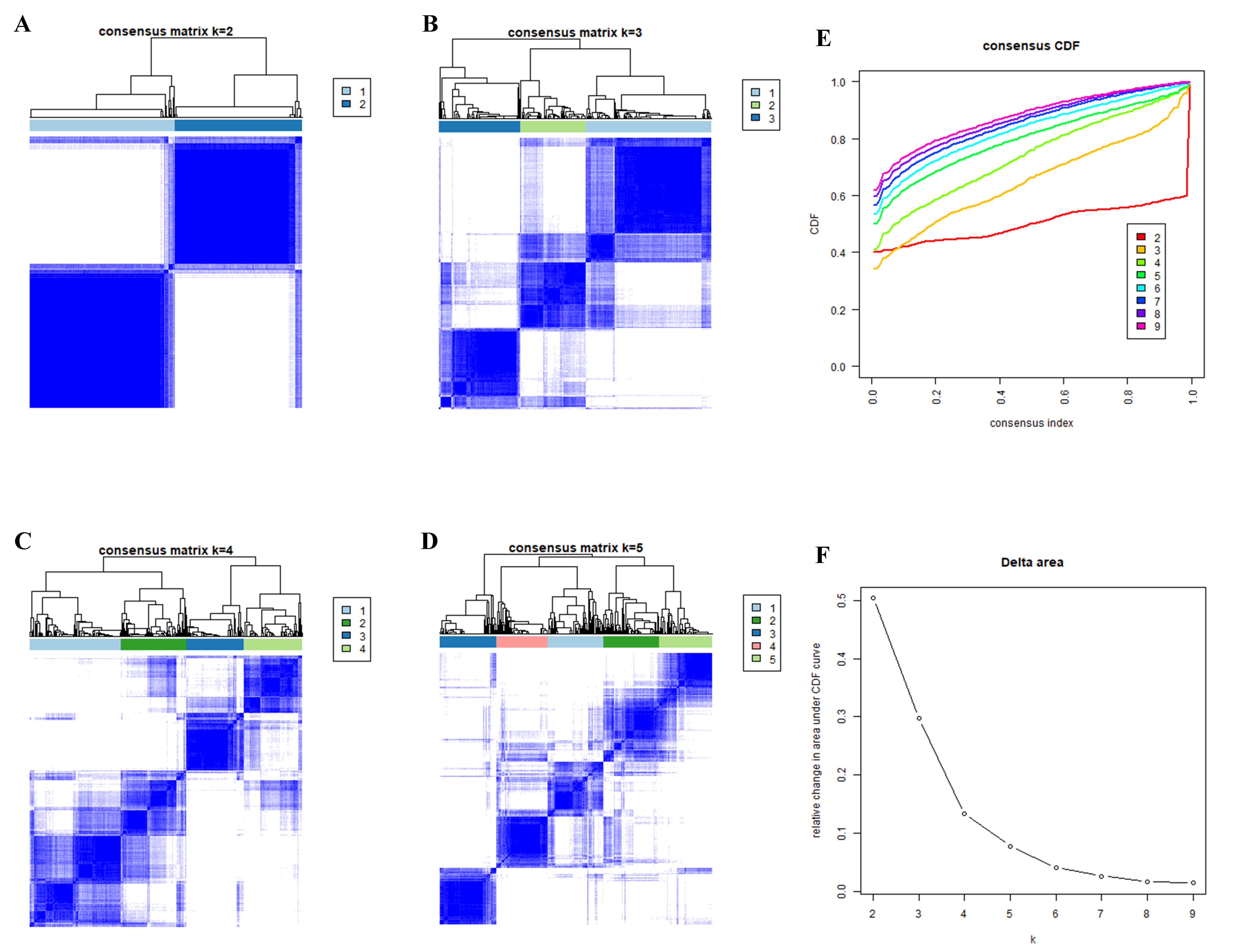

Supplement: Supplementary file 3 [file Image4.TIF]

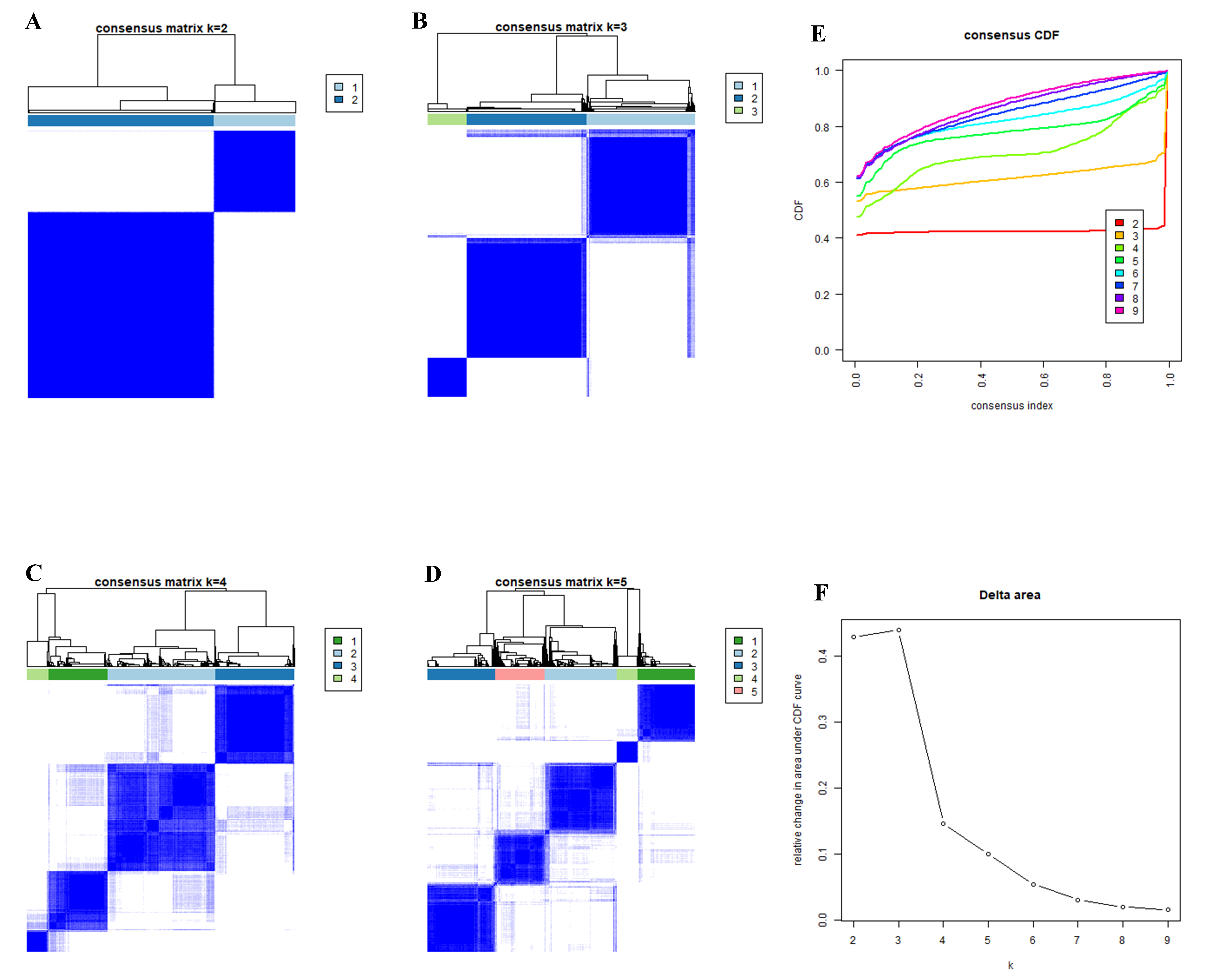

Supplement: Supplementary file 5 [file Image2.TIF]

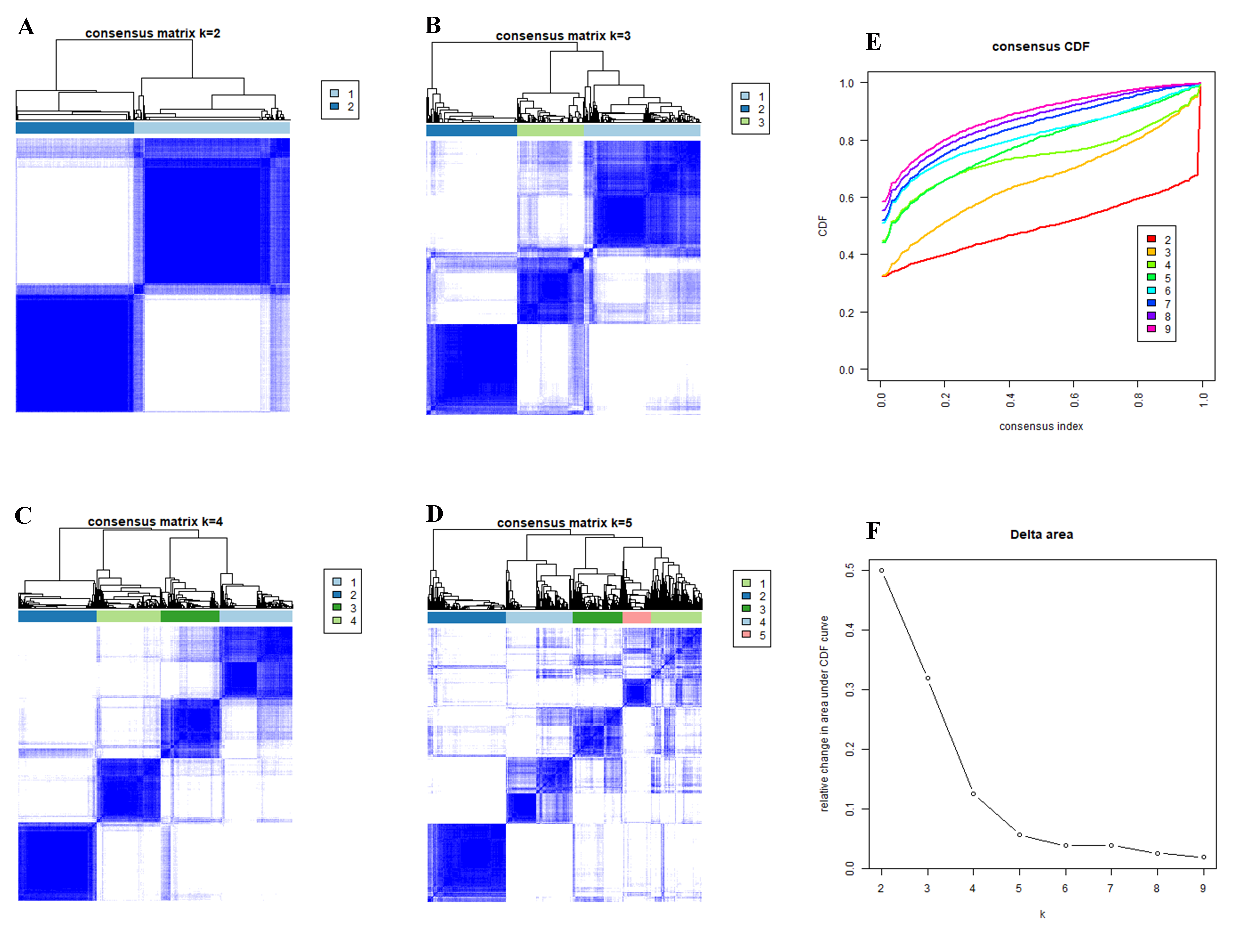

Supplement: Supplementary file 6 [file Image1.TIF]

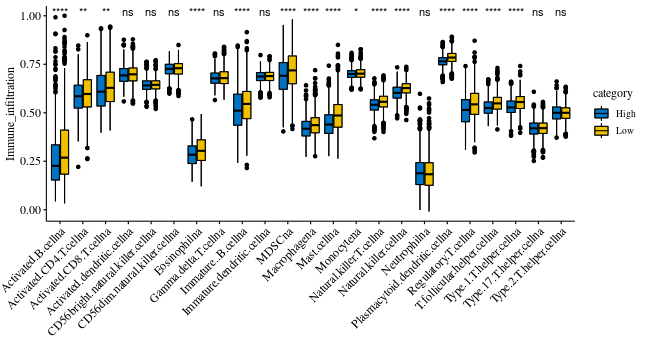

Supplement: Supplementary file 10 [file Image3.PNG]
